# Efficacy of vancomycin-releasing biodegradable poly(lactide-*co*-glycolide) antibiotics beads for treatment of experimental bone infection due to *Staphylococcus aureus*

**DOI:** 10.1186/s13018-016-0386-x

**Published:** 2016-04-27

**Authors:** Steve W. N. Ueng, Song-Shu Lin, I-Chun Wang, Chuen-Yung Yang, Ru-Chin Cheng, Shih-Jung Liu, Err-Cheng Chan, Cheng-Fen Lai, Li-Jen Yuan, Sheng-Chieh Chan

**Affiliations:** Department of Orthopaedic, Chang Gung Memorial Hospital, Linkou, Taiwan; Department of Orthopaedic, Chang Gung Memorial Hospital, Keelung, Taiwan; Department of Nuclear Medicine, Chang Gung Memorial Hospital, Keelung, Taiwan; Department of Mechanical Engineering, Chang Gung University, Taoyuan, Taiwan; Department of Medical Biotechnology and Laboratory Science, Chang Gung University, Taoyuan, Taiwan; Laboratory Animal Center, Chang Gung Memorial Hospital, Keelung, Taiwan; Bone and Joint Research Center, Chang Gung Memorial Hospital, Linkou, Taiwan; Department of Orthopaedic Surgery, Chang Gung Memorial Hospital, No 5, Fu-Hsing Street 333, Kweishan, Taoyuan Taiwan

**Keywords:** Bone infection, PLGA, ^18^F-FDG PET, Vancomycin

## Abstract

**Background:**

Clinical experience and animal studies have suggested that positron emission tomography (PET) using fluorine-18-labeled fluorodeoxyglucose (^18^F-FDG) may be promising for imaging of bone infections. In this study, we aimed to establish the accuracy of ^18^F-FDG PET scanning for monitoring the response to poly(lactide-*co*-glycolide) (PLGA) vancomycin beads for treatment of bone infection.

**Methods:**

PLGA was mixed with vancomycin and hot-compress molded to form antibiotic beads. In vitro, elution assays and bacterial inhibition tests were employed to characterize the released antibiotics. In vivo, cylindrical cavities were made in six adult male New Zealand white rabbits, and *Staphylococcus aureus* or saline was injected into the cavity to create a bone infection. After 2 weeks, the infection was confirmed by bacterial cultures, and the defect was filled with PLGA vancomycin beads. The treatment response was monitored by ^18^F-FDG PET.

**Results:**

The biodegradable beads released high concentrations of vancomycin (well above the breakpoint sensitivity concentration) for treatment of bone infection. In bacterial inhibition tests, the diameter of the sample inhibition zone ranged from 6.5 to 10 mm, which was equivalent to 12.5–100 % relative activity. ^18^F-FDG PET results showed that uncomplicated bone healing was associated with a temporary increase in ^18^F-FDG uptake at 2 weeks, with return to near baseline at 6 weeks. In the infected animals, localized infection resulted in intense continuous uptake of ^18^F-FDG, which was higher than that in uncomplicated healing bones. Bone infection was confirmed with positive bacterial cultures. In vancomycin-treated animals, data showed rapidly decreasing amounts of ^18^F-FDG uptake after treatment.

**Conclusions:**

In vitro and in vivo analyses showed that the use of biodegradable PLGA vancomycin beads successfully eradicated *S. aureus* infection in damaged bone.

## Background

Despite advances in surgical techniques and the availability of newly developed antibiotics, bone infections after surgical procedures and trauma continue to be a difficult problem for surgeons [1, 2]. Typically, operative debridement and antibiotic therapy are preferred treatments for bone infection [3]. Although intravenous route of antibiotic injection produces adequate antibiotic concentration in the blood, recurrence of infection, the high cost of the antibiotics, and poor patient compliance are still problems. Osseous defects after debridement can be reconstructed using various techniques, such as cancellous bone grafting, free microvascular bone transfer, and distraction osteogenesis [1, 4]. However, substantial morbidity is associated with harvesting of bone grafts and bone transplantation. Currently, the standard treatment remains a combination of surgical intervention and administration of an effective local antibiotic therapy [1, 5]. For antibiotic treatment, incorporation of antibiotics into polymethyl methacrylate (PMMA) [6, 7], a concept that was originally developed by Buchholz et al., is commonly used [8]. Antibiotic-impregnated PMMA beads provide a high local concentration of the drug and have advantages over intravenous injection in that the beads yield fewer systemic complications and allergic reactions. However, PMMA beads require a second operation for removal after prolonged implantation [1, 9].

Various types of carrier materials have been used based on their ability to achieve sustained bactericidal concentrations of the antibiotic [3, 10–13]. A biodegradable carrier as a slow release system for delivering antibiotics may be better than PMMA beads and intravenous antibiotics in several ways. First, biodegradable beads provide long-term administration of a bactericidal concentration of the antibiotic, and the biodegradability of the beads can be varied to treat many types of infections. Second, because the biodegradable beads dissolve, there is no need for surgical removal and soft tissue reconstruction. Poly(lactide-*co*-glycolide) (PLGA) copolymer is a promising biodegradable material that is nontoxic, elicits a minimal inflammatory response, and can be absorbed with no accumulation in vital organs. Garvin et al. described the use of polyglycolide beads loaded with gentamicin for effective treatment of tibial *Staphylococcus aureus* osteomyelitis in a canine model [14]. Ueng et al. developed a hot-compress molded method to manufacture PLGA copolymer antibiotic beads and achieved sustained in vitro antibiotic release for more than 30 days [11, 12]. In addition, Ueng et al. showed in vivo antibiotic release for 56 days from PLGA copolymer antibiotic beads in a rabbit model [15]. However, the efficacy of PLGA vancomycin beads for treatment of experimental bone infection due to *S. aureus* has never been reported.

Fluorine-18-fluoro-2-deoxy-D-glucose positron emission tomography combined with computed tomography (^18^F-FDG PET/CT) is a cutting-edge functional imaging method that provides images with higher resolution and concomitant anatomical information. ^18^F-FDG PET/CT has been used as a diagnostic imaging modality for detection of prosthetic joint infection [16, 17], Alzheimer’s disease [18], inflammatory and infectious vascular disease [19], and tumors [20]. Postsurgical infection is one of the most prevalent and challenging complications faced by orthopedic surgeons and patients, and clinical experience suggests that ^18^F-FDG PET/CT may be promising for imaging of bone infections [21–24]. Previous animal studies have shown that ^18^F-FDG uptake is increased in the context of bacterial infections [25–29]. ^18^F-FDG uptake is also significantly higher in the osteomyelitic region than in healing bone [28].

In the present study, we evaluated the release of vancomycin from PLGA vancomycin beads and examined the relative activity of the released antibiotics in vitro. In addition, we examined the use of ^18^F-FDG PET/CT for quantitative monitoring of the response to PLGA vancomycin bead treatment in a model of bone infection.

## Methods

### Development of a biodegradable bead for antibiotics

A biodegradable antibiotic bead was developed using PLGA copolymer and vancomycin (Abbott Laboratories, North Chicago, IL, USA). The PLGA copolymers used were poly(D,L)-lactide-co-glycolide with a ratio of 50:50 and an intrinsic viscosity of 0.4 (Boehringer Ingelheim KG, Germany). All copolymers were available in powder form with particle size ranges from 100 to 200 mm. The copolymer and vancomycin were premixed using a dry mixer (copolymer to vancomycin ratio of 5:1). The mixture was molded into 5-mm diameter beads by a compression molding machine at 55 ° C [12, 15].

### In vitro elution assay

An in vitro elution method was employed to determine the release characteristics of antibiotics from the beads. A phosphate-buffered saline (PBS, pH 7.4) was used as the dissolution medium. The beads were placed in glass test tubes with a volume of 1 mL PBS. All tubes were incubated at 37 °C. The dissolution medium was collected and analyzed at every 24-h interval. Fresh PBS (1 mL) was then added for the next 24-h period, and this procedure was repeated until the capsule was fully dissolved. The antibiotic concentrations in buffer for the elution studies were determined by a high-performance liquid chromatography (HPLC) assay standard curve for vancomycin. The HPLC analyses were conducted on a Walters 600 Multi-solvent Delivery System. The column used for separation of the antibiotics was a SYMMETRY C8, 3.9 cm × 150 mm HPLC column (Waters). The mobile phase contained 0.01 mol heptanesulphonic acid (Fisher Scientific UK Ltd.) and acetonitrile (Mallinckrodt, USA) (85/15, *v*/*v*). The absorbency was monitored at 280 nm, and the flow rate was 1.4 mL/min. All samples were assayed in triplicate, and sample dilutions were performed to bring the unknown concentrations into the range of the assay standard curve. A calibration curve was made for each set of the measurements (correlation coefficient >0.99).

### Activities of antibiotics

The minimum inhibitory concentration (MIC) of vancomycin to *S. aureus* (ATCC 259523) was determined using an antibiotic tube dilution method in Cation Supplemented Mueller-Hinton Broth (Difco Laboratories, Detroit, MI). Vancomycin was diluted serially twofold in tubes containing 0.5 mL of the cation-supplemented Mueller-Hinton broth. The *S. aureus* inocula for each series of tubes was 0.5 mL of an overnight culture containing 5.0 × 10^5^ colony-forming units/mL. The MIC was considered to be the lowest concentration of antibiotic that prevented turbidity after 24 h of incubation at 37 °C. The antibiotic concentrations at the transition point between bacterial killing and resistance to the antibiotic (the breakpoint sensitivity limit) for vancomycin were determined by tube dilution sensitivities.

The relative activity test of vancomycin to *S. aureus* (ATCC65389) was determined using an antibiotic disk diffusion method in Nutrient Broth (beef extract, peptone, Difco Laboratories). The eluent of the capsules was tested up to 36 days. Each sample was first diluted or concentrated to 50 mg/mL. Eight microliters of the buffer sample from each daily buffer sample was pipetted onto 6-mm absorption disks. The disks were placed on the nutrient agar plates that were seeded with a layer of *S. aureus*, and the zones of inhibition were measured with a micrometer after 16–18 h of incubation at 35 °C. The equation and other relevant items for relative activity were as follows: relative activity (%) = (the diameter of sample inhibition zone − the diameter of disk)/(the diameter of maximum inhibition zone − the diameter of disk).

### Animal model of bone defect

Six adult male New Zealand white rabbits weighing 2.5–3.4 kg were used. All animal procedures were reviewed and approved by the Institutional Animal Care and Use Committee of the Chang Gung Memorial Hospital. The methods were carried out in accordance with the approved guidelines.

### Induction of infection (first stage of surgery)

A cylindrical bony cavity (15 mm × 12 mm × 10 mm) was surgically made using a round bur and obliterated with a customized PMMA spacer at the left and right distal femur of each rabbit. The bone cement acted as a foreign body to conduct infection. Subsequently, 0.1 mL of 1 × 10^5^ colony-forming units/mL of *S. aureus* was withdrawn by a pipette tip and directly applied to the space surrounding the bone cement (three rabbits), and 0.1 mL of sterile saline was applied to the corresponding space of control group (three rabbits). The periosteal and fascial layers were closed over the cortical defect.

### Debridement (second stage of surgery)

Two weeks after the initial surgery, all animals underwent the second stage of surgery. Using the previous surgical approach, the defect area was exposed and swab cultures were taken to confirm that the induction of staphylococcal bone infection had been successful. The biodegradable PLGA vancomycin beads were implanted into the cavities for infection therapy. The swab specimens were cultured for 20 h at 35 °C in blood agar plates. The bone cement was removed and separately cultured for 4 days at 35 °C on brain heart infusion solution (BBL; Becton Dickinson).

### ^18^F-FDG PET/CT

^18^F-FDG PET was performed for each animal at 2 weeks after the second stage of surgery. A mean of 96 MBq of ^18^F-FDG (range, 89–100 MBq) was injected into the ear artery. The animals were not fasted before ^18^F-FDG administration. PET/CT scanning was performed 40 min after the injection of ^18^ F-FDG with the GE DSTE PET/CT scanner that was equipped with a three-dimensional (3D) mode image acquisition system. The PET acquires 47 contiguous slices with an axial field of view of 70 cm. The slice thickness of the scanner is 3.75 mm, and the spatial resolution was 6 mm in full width at half maximum in the center of the field of view. The CT was performed using a 16-slice helical CT. The CT scan data were collected at an auto mAs and 120 kV. After the CT scan, an emission scan was obtained from knee at a rate of 3 min per bed. Attenuation-corrected PET images using the CT data were reconstructed using an ordered subset expected maximization algorithm (20 subsets, 2 iterations).

### PET data analysis

Quantitative analysis was performed on the circular regions of interest (ROIs) area of the right and left distal femur. ^18^F-FDG accumulation was reported as the standardized uptake value (SUV), which was calculated as the radioactivity of the ROI divided by the relative injected dose, expressed per kilogram of body weight.

## Results

### Development of biodegradable vancomycin beads

**Fig. 1:**
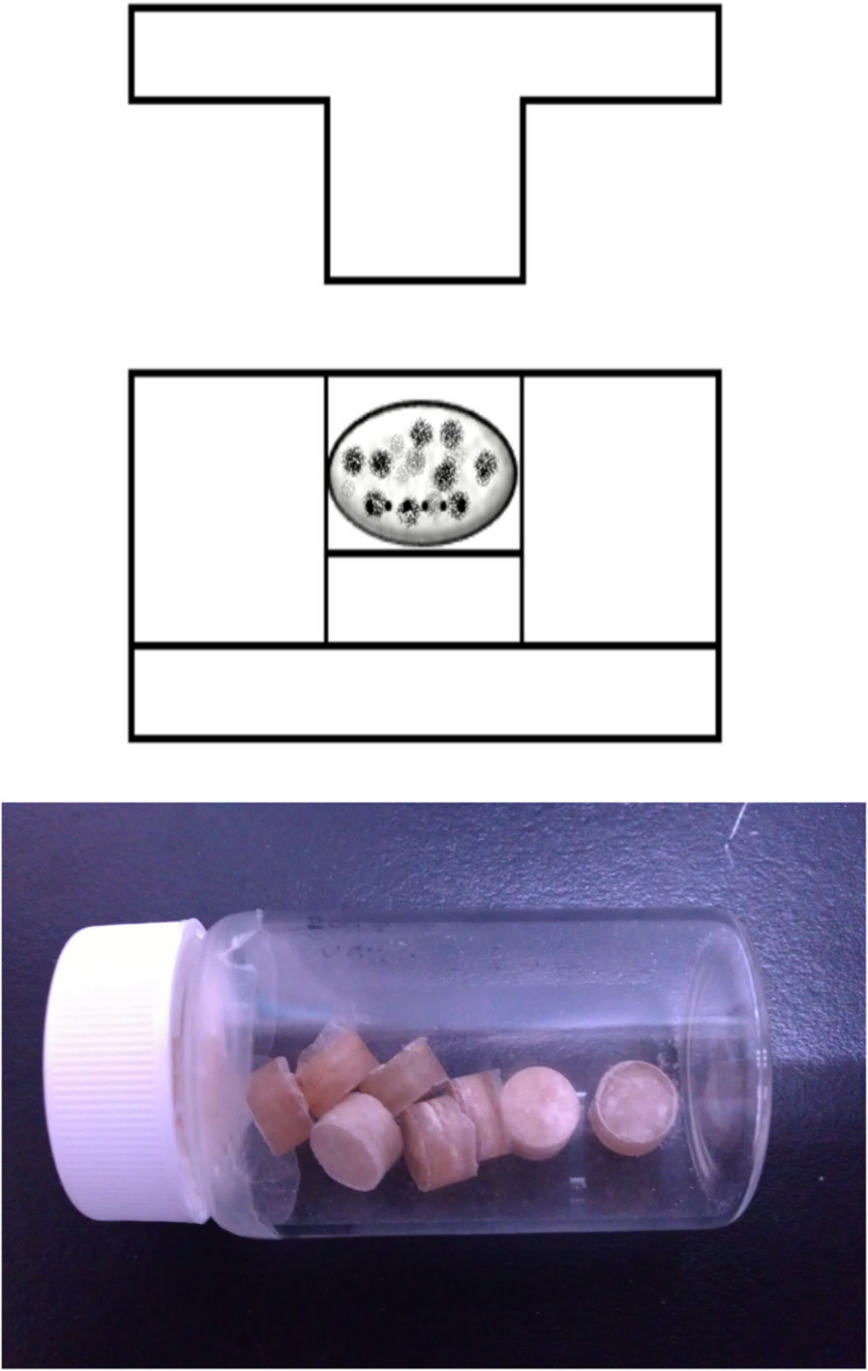
Development of biodegradable vancomycin beads. The PLGA vancomycin mixture was hot-compressed into a vancomycin bead of 5 mm in diameter

The PLGA vancomycin mixture was hot-compressed into a vancomycin bead of 5-mm diameters, as shown schematically in Fig. 1.

### In vitro elution assay

**Fig. 2:**
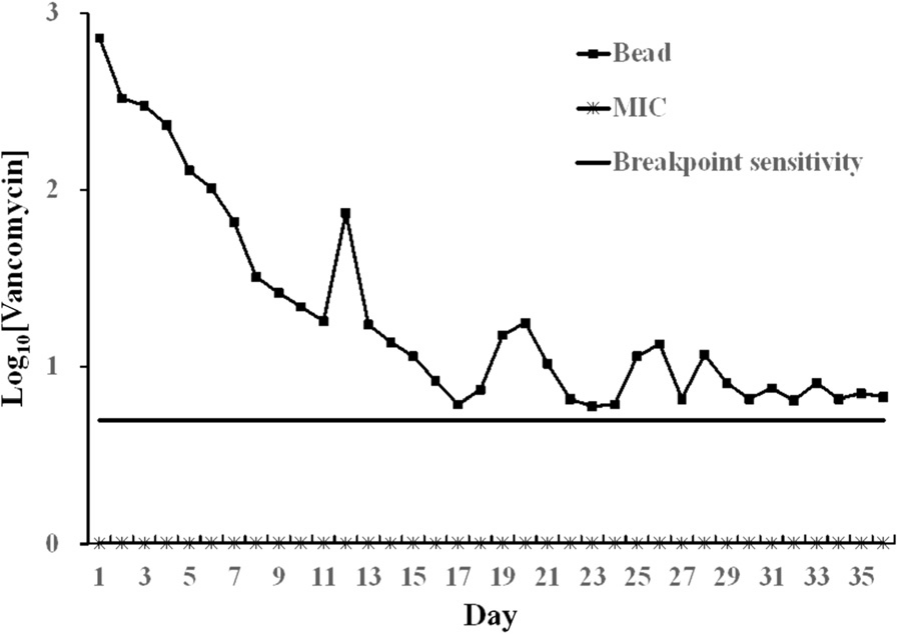
Release curves of biodegradable vancomycin beads. Each *point* represents three samples. The MIC and breakpoint sensitivity of vancomycin for *S. aureus* were 1.01 and 5 mg/L

The release curves of vancomycin from the PLGA beads are shown in Fig. 2. Three samples were performed for each test. The mean vancomycin concentrations on days 1, 2, 5, 10, 15, 20, 25, 30, and 35 from the beads were 724.4, 331.1, 128.8, 21.9, 11.5, 17.8, 11.5, 6.6, and 7.1 mg/L, respectively. Gradual release of vancomycin was observed from the biodegradable beads over the entire 40 days, remaining above the breakpoint sensitivity level through day 36. However, vancomycin release was the most obvious during the first 48 h. The MIC and breakpoint sensitivity of vancomycin for *S. aureus* were 1.01 and 5 mg/L, respectively.

### Relative activity test of eluted vancomycin

**Fig. 3:**
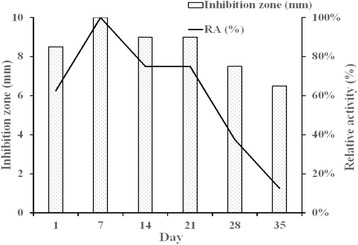
Results of relative activity and the diameter of the inhibition zone. Each *point* represents three samples. The diameters of the sample inhibition zones ranged from 6.5 to 10 mm and the relative activity ranged from 12.5 to 100 %

The resulting values for relative activity and the diameter of the inhibition zone for beads are shown in Fig. 3. Each point represents three samples. The diameters of the sample inhibition zones ranged from 6.5 to 10 mm, and the relative activity ranged from 12.5 to 100 %.

### Confirmation of staphylococcal infection and vancomycin treatment

**Fig. 4:**
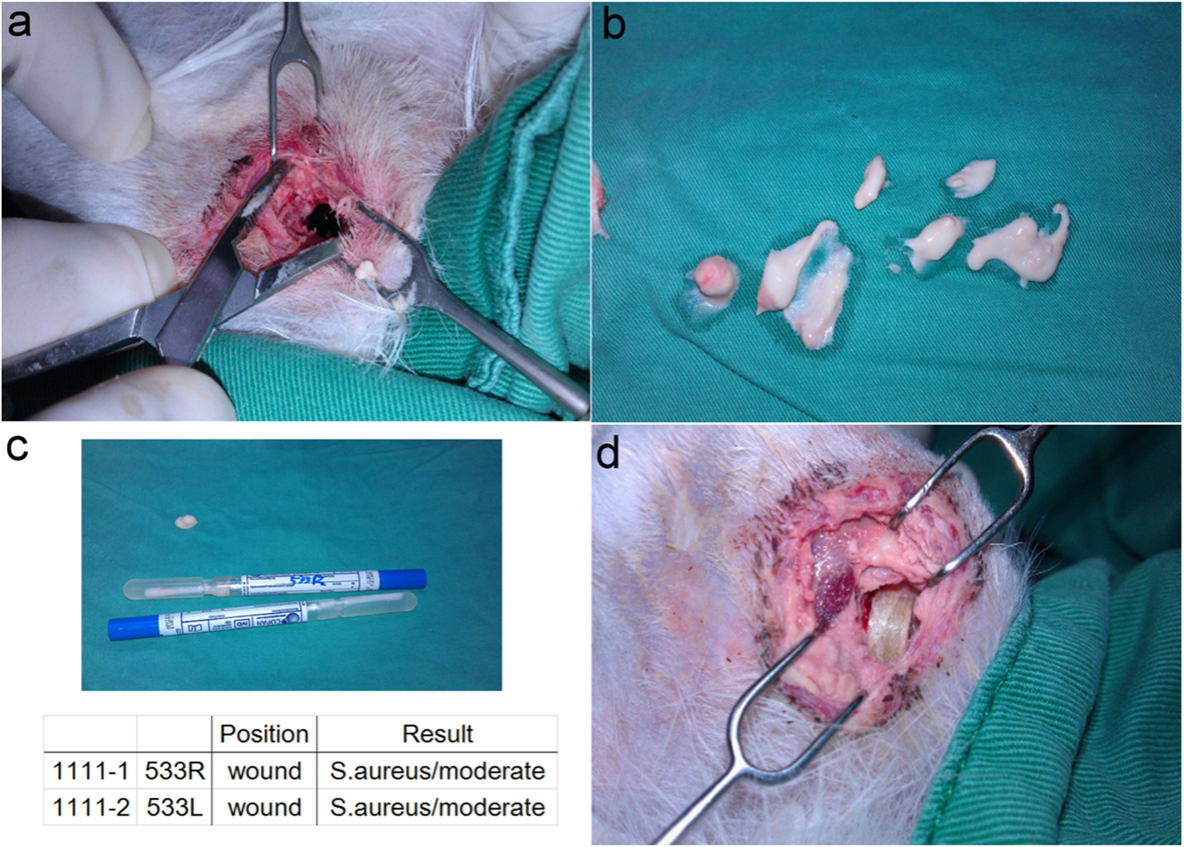
Confirmation of staphylococcal infection and vancomycin treatment. In the infected animals, bone infection was confirmed with positive bacterial cultures during the debridement (**a–c**) and the defect was filled with a PLGA vancomycin bead (**d**)

In the infected animals, bone infection was confirmed with positive bacterial cultures during the debridement (Fig. 4a–c), and the defect was filled with a PLGA vancomycin bead (Fig. 4d). In the control group, none of the corresponding cultures were positive.

### ^18^F-FDG PET/CT imaging

**Fig. 5:**
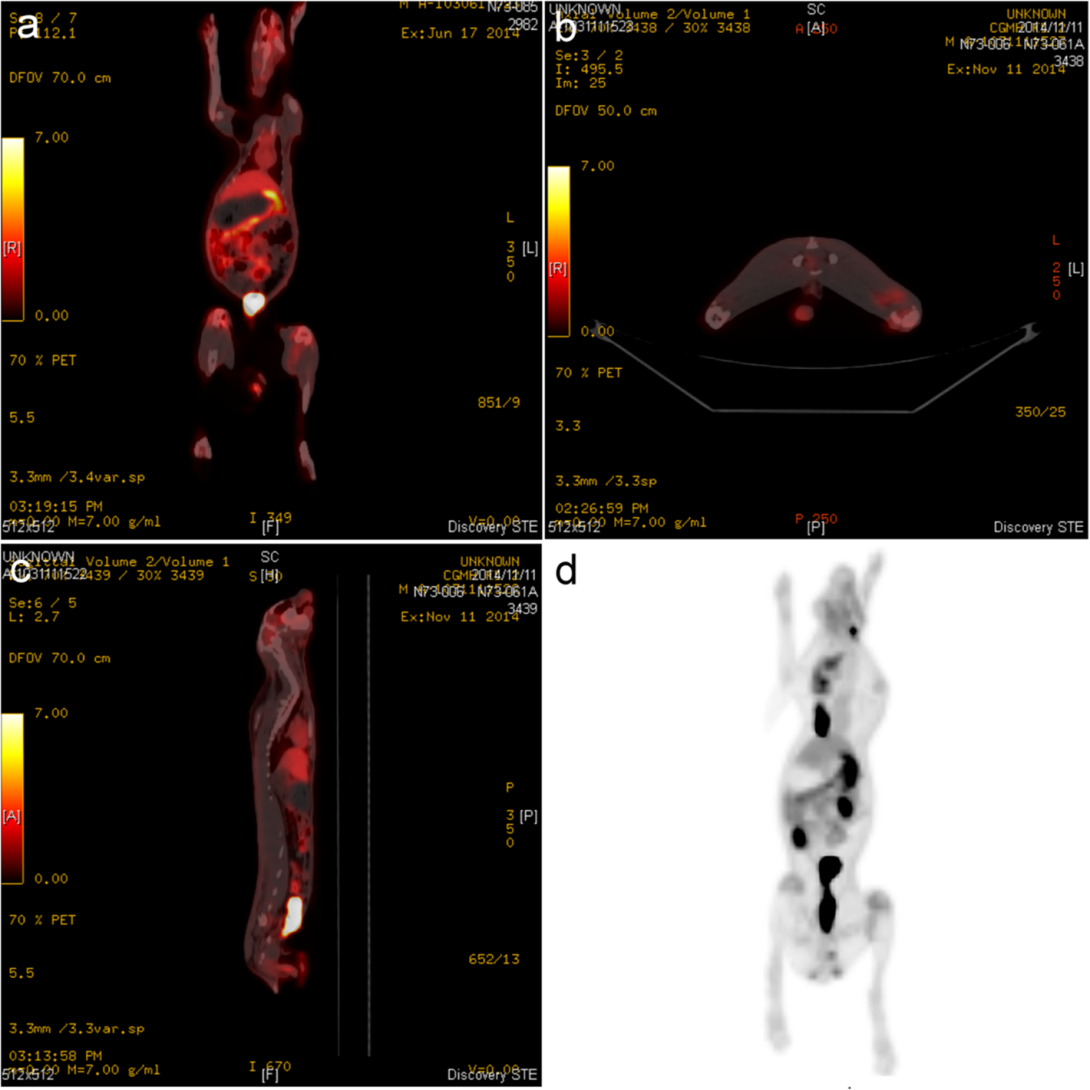
^18^F-FDG PET/CT imaging. Coronal (**a**), transaxial (**b**), and sagittal (**c**) ^18^F-FDG PET/CT images. (**d**) PET image for quantitative analysis

**Fig. 6:**
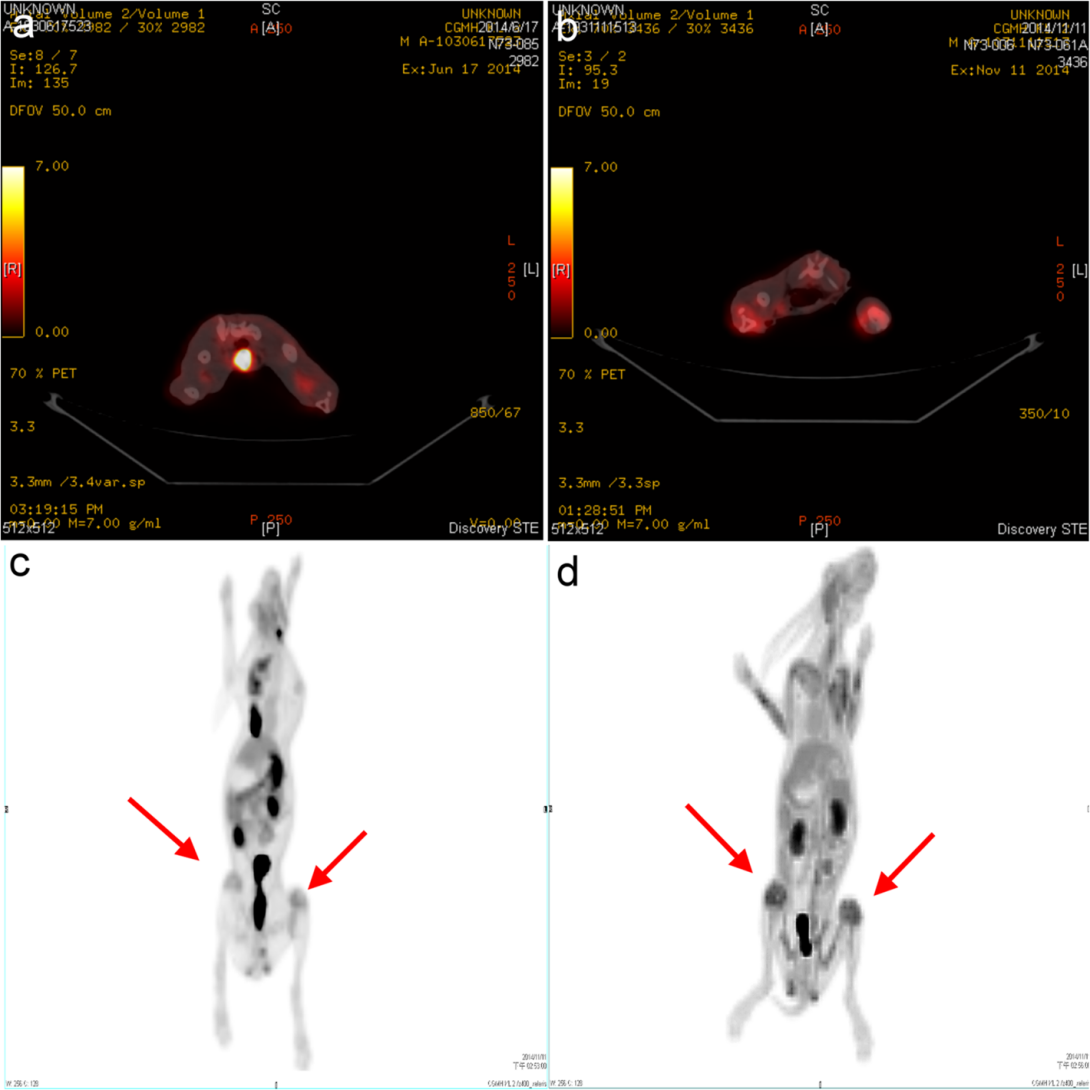
^18^F-FDG PET/CT images in the control (**a**, **c**) and infection groups (**b**, **d**). Localized infection resulted in an intense continuous uptake of ^18^F-FDG (**b**, **d**) which was higher than that of healing bones in control group (**a**, **c**)

**Fig. 7:**
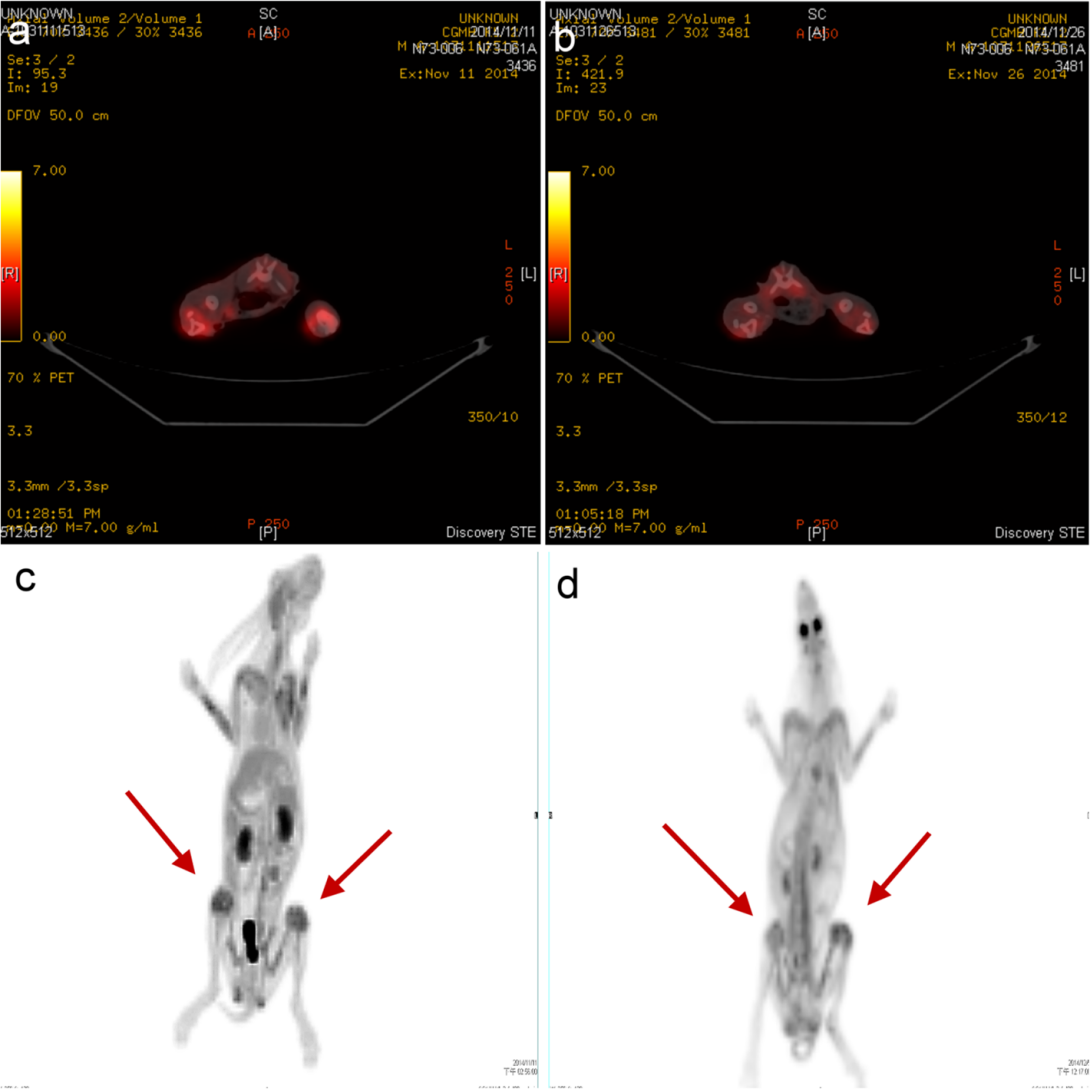
^18^F-FDG PET/CT images in the infection (**a**, **c**) and vancomycin-treated groups (**b**, **d**). Compare to the initial deep infection (**a**, **c**), PET images showed rapidly decreasing amounts of ^18^F-FDG intensity after 2 weeks of vancomycin beads treatment in infected bone regions (**b**, **d**)

**Fig. 8:**
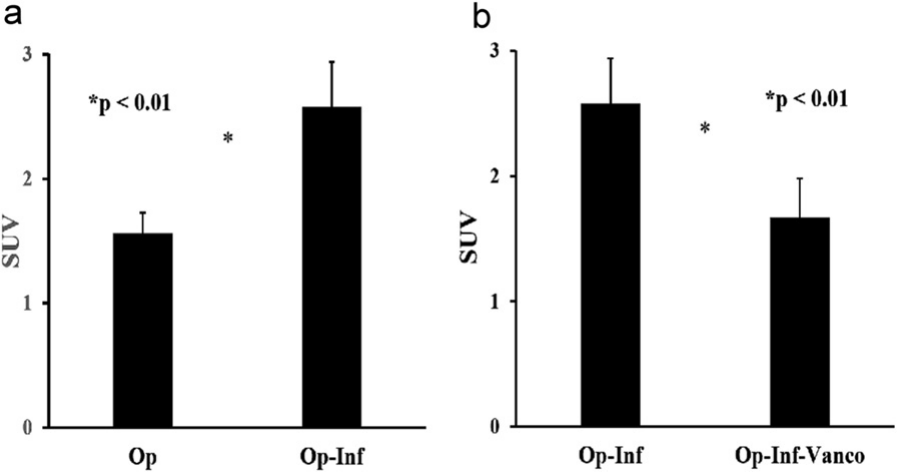
Quantitative analysis of PET image. **a** The infection group showed a significantly higher mean SUV than that of the control group (*p* < 0.01). (**b**) Mean SUV data showed a significantly lower value after vancomycin beads treatment (*p* < 0.01). *Op* operation, *Inf* infection, *Vanco* vancomycin

Coronal (Fig. 5a), transaxial (Fig. 5b), and sagittal (Fig. 5c) ^18^F-FDG PET/CT images were acquired. ^18^F-FDG PET image for quantitative analysis is shown in Fig. 5d. There were significant differences in ^18^F-FDG uptake between the experimental and control groups. Two weeks after the initial surgery, localized infection resulted in an intense continuous uptake of ^18^F-FDG (Fig. 6b, d), which was higher than that of the healing bones in the control group (Fig. 6a, c). Compared with the initial deep infection (Fig. 7a, c), PET images showed rapidly decreasing amounts of ^18^F-FDG intensity after 2 weeks of vancomycin beads treatment in the infected bone regions (Fig. 7b, d). In the mean SUV results, the infection group showed a significantly higher mean SUV than the control group (control group vs. infection group: 1.56 ± 0.17 vs. 2.58 ± 0.36, *p* < 0.01, *n* = 6; Fig. 8a). The use of biodegradable PLGA vancomycin beads was successful for eradication of the *S. aureus* pathogen from bone (pretreatment vs. posttreatment: 2.58 ± 0.36 vs. 1.67 ± 0.30, *p* < 0.01, *n* = 6; Fig. 8b).

## Discussion

In the present study, in vitro data showed that the biodegradable beads released high concentrations of vancomycin during the time needed to treat bone infection. The diameter of the sample inhibition zone ranged from 6.5 to 10 mm, which was equivalent to 12.5–100 % relative activity in the bacterial inhibition test. In vivo results showed that localized infection resulted in intense continuous uptake of ^18^F-FDG, which was higher than that in uncomplicated healing bones. Bone infection was confirmed with positive bacterial cultures. In the vancomycin-treated animals, data showed rapidly decreasing amounts of ^18^F-FDG uptake after treatment.

Bacterial infections in surgery can be devastating and are often associated with considerable morbidity and poor functional outcomes [30]. The management of postoperative infection requires surgical debridement, the removal of all necrotic tissue and implants, and the administration of systemic antibiotics. Nevertheless, antibiotic treatment may be inadequate or ineffective in patients with poorly vascularized infected tissues [31]. Delivering local antibiotics by using antibiotic-impregnated biodegradable carriers has been proposed to provide a sustained release of antibiotics to infected areas, replacing intravenous antibiotics infusion. The possibility of PLGA copolymer being a biodegradable antibiotic carrier has been studied for years. In most previous studies, the antibiotic was microencapsulated in a PLGA bead with a high molecular weight (molecular weight 3.26 × 10^4^ Da), a high percentage of the poly(lactic acid) ratio (70:30), a high antibiotic loading dosage (50 %), and a high formation temperature (110 °C) [32–35]. The present study used a hot-compress molded method with a low molecular weight (molecular weight 5000 Da vs. 3.26 × 10^4^ Da), a low ratio of poly(lactic acid) ratio copolymer (50:50 vs. 70:30), a low antibiotic loading dosage (16.6 vs. 50 %), and a low formation temperature (55 vs. 110 °C) to form a biodegradable antibiotic bead delivery system. In this study, the authors achieved 36 days of a sustained release of antibiotic from this antibiotic delivery system in vitro (Figs. 2 and 3) which is adequate for treatment of posttraumatic osteomyelitis (3–6 weeks) [3, 32].

Based on our in vitro results, we next monitored the treatment response of PLGA vancomycin beads in the infection regions in vivo. ^18^F-FDG, the most commonly used PET tracer for diagnosis of infection [36], has been shown to localize autoradiographically in the regions with the highest numbers of macrophages and leukocytes [26]. The use of ^18^F-FDG PET to image bacterial infections is based on the intensive use of glucose by macrophages and leukocytes [26]. Fluoro-D-deoxyglucose is transported into cells via glucose transporters and phosphorylated by hexokinase to ^18^F-2′-FDG-6 phosphate but is not metabolized. The degree of cellular FDG uptake is related to the cellular metabolic rate and to the number of glucose transporters [37]. In activated cells, such as neutrophils, lymphocytes, monocytes, and macrophages, both the number and expression of glucose transporters are increased, and the affinity of deoxyglucose for these transporters is also increased [38–40]. Although the early stages of bone healing, involving an inflammatory phase with a highly activated state of cell metabolism and glucose consumption may mimic infection on PET images [41, 42]. However, the localized infection resulted in an intense continuous uptake of ^18^F-FDG (Fig. 6b, d), which was higher than that of the healing bones in the inflammatory phase (Fig. 6a, c) in this study. Our results consist with previous study demonstrating that bone infection could be distinguished from bone healing by ^18^F-FDG PET analysis [28].

Previous studies have shown that localized infection by *S. aureus* pathogen resulted in an intense continuous uptake of ^18^F-FDG over 3 weeks in an untreated-infection group [28, 43]. In the present study, PET images showed decreasing amounts of ^18^F-FDG intensity in infected bone regions after bead treatment for 2 weeks (Fig. 7b, d). The mean SUV data also showed a significantly lower value after bead treatment (Fig. 8). Thus, our results showed that the use of biodegradable PLGA vancomycin beads was successful for eradication of the *S. aureus* pathogen from bone.

The rabbit model of *S. aureus* osteomyelitis has been used to explore the diagnostic efficacy of ^18^F-FDG PET scan. Lankier et al. showed that foreign-body-associated infection in the rabbit tibia caused by *Staphylococcus epidermidis* results in lower ^18^F-FDG uptake than pyogenic *S. aureus* infections [44]. Koort et al. showed that bone healing is associated with a temporary increase in ^18^F-FDG uptake, followed by a returned to baseline [28]. In contrast, localized osteomyelitis resulted in a significantly higher, continuous uptake of ^18^F-FDG. Similar results have been reported in a clinical study [45]. ^18^F-FDG PET/CT scanning used for monitoring therapeutic response to antimicrobials in experimental osteomyelitis also has been reported. Koort et al. showed the value of ^18^F-FDG PET for quantitative monitoring of the ciprofloxacin bone defect filler treatment response in bone infections [25]. Chatziioannou et al. showed the value of ^18^F-FDG PET for quantitative monitoring of the daptomycin treatment response in infected tibia [43].

Our study has several limitations. Our experiments were limited to a small number of animals in each group, and it could be argued that the differences we observed were even more significant given the small size of the experimental groups. Additionally, given the limited volume of the bony cavity, only a single dosage of vancomycin bead was used. Although this study was not designed to determine the optimal dosage and duration of vancomycin for treatment of bone infection, it establishes a conceptual framework by which to develop a large animal using dogs or goats. In addition, one of the inherent limitations of this study is that we do not know quite well yet how well the results of this study translate clinically.

In summary, ^18^F-FDG PET/CT imaging technology is a sensitive and specific tool in the diagnosis of experimental bon infection and in monitoring the therapeutic effects of PLGA vancomycin beads. The present study offers a biodegradable antibiotics bead to meet clinical requirements for the treatment of bone infection.

## Conclusions

Taken together, the in vitro and in vivo results showed that the use of biodegradable PLGA vancomycin beads was successful for eradication of the *S. aureus* pathogen from bone. These results may have important clinical applications.

## Ethics approval and consent to participate

All animal procedures were reviewed and approved by the Institutional Animal Care and Use Committee of the Chang Gung Memorial Hospital.

## Abbreviations

CT: computed tomography
F-FDG: fluorine-18-labeled fluorodeoxyglucose
HPLC: high-performance liquid chromatography
MIC: minimum inhibitory concentration
PBS: phosphate-buffered saline
PET: positron emission tomography
PLGA: poly(lactide-*co*-glycolide)
PMMA: polymethyl methacrylate
SUV: standardized uptake value

## References

[1] Ueng SWN, Wei FC, Shih CH (1997). Management of large infected tibial defect with antibiotic beads local therapy and staged fibular osteoseptocutaneous free transfer. J Trauma.

[2] Gustilo RB, Mendoza RM, Williams DN (1984). Problems in the management of type III open fracture. J Trauma.

[3] Mader JT, Calhoun J, Cobos J (1997). In vitro evaluation of antibiotic diffusion from antibiotic-impregnated biodegradable beads and polymethylmethacrylate beads. Antimicrob Agents Chemother.

[4] Patzakis MJ, Mazur K, Wilkins J (1993). Septopal beads and autogenous bone grafting for bone defects in patients with chronic osteomyelitis. Clin Orthop Relat Res.

[5] Cierny G, Mader JT (1985). A clinical staging system for adult osteomyelitis. Contemp Orthop.

[6] Greene N, Holtom PD, Warren CA (1998). In vitro elution of tobramycin and vancomycin PMMA beds and spacers from Simplex and Palacos. Am J Orthop.

[7] Holtom PD, Warren CA, Greene NW (1998). Relation of surface area to in vitro elution characteristics of vancomycin-impregnated PMMA spacers. Am J Orthop.

[8] Buchholz HW, Engelbrecht H (1970). Depot effects of various antibiotics mixed with Palacos resins. Chirurg.

[9] Henry SL, Seligson D, Mangino P (1991). Antibiotic-impregnated beads. Part I: bead implantation versus systemic therapy. Orthop Rev.

[10] Ueng SWN, Lee MS, Lin SS (2007). Development of a biodegradable alginate carrier system for antibiotics and bone cells. J Orthop Res.

[11] Lin SS, Ueng SW, Liu SJ (1999). Development of a biodegradable antibiotic delivery system. Clin Orthop.

[12] Liu SJ, Ueng SW, Chan EC (1999). In vitro elution of vancomycin from biodegradable beads. J Biomed Mater Res.

[13] Shinto Y, Uchida A, Korkusaz F (1992). Calcium hydroxyapatite ceramic used as a drug delivery system for antibiotics. J Bone Joint Surg.

[14] Garvin KL, Miyano JA, Robinson D (1994). Polylactide/polyglycolide antibiotic implants in the treatment of osteomyelitis: a canine model. J Bone Joint Surg.

[15] Ueng SWN, Yuan LJ, Lee N (2002). In vivo study of hot compressing molded 50:50 poly (DL-lactide-co-glycolide) antibiotic beads in rabbits. J Orthop Res.

[16] Gemmel F, Van den Wyngaert H, Love C (2012). Prosthetic joint infections: radionuclide state-of-the-art imaging. Eur J Nucl Med Mol Imaging.

[17] van der Bruggen W, Bleeker-Rovers CP, Boerman OC (2010). PET and SPECT in osteomyelitis and prosthetic bone and joint infections: a systematic review. Semin Nucl Med.

[18] Ota K, Oishia N, Ito K (2015). Effects of imaging modalities, brain atlases and feature selection on prediction of Alzheimer’s disease. J Neurosci Methods.

[19] Chrapko B, Chrapko M, Nocuń A (2016). Role of 18F-FDG PET/CT in the diagnosis of inflammatory and infectious vascular disease. Nucl Med Rev.

[20] Scialpi M, Palumbo I, Gravante S (2016). Multi-detector row CT fusion imaging in oncologic patients: preliminary results. Radiology.

[21] Fathinul F, Nordin A (2010). ^18^F-FDG PET/CT as a potential valuable adjunct to MRI in characterising the Brodie's abscess. Biomed Imaging Interv J..

[22] Beslic N, Heber D, Walter Lipp R (2015). Metabolic pattern of asymptomatic hip prosthesis by ^18^F-FDG–positron-emission-tomography. Iran J Radiol.

[23] Gemmel F, Rijk PC, Collins JM (2010). Expanding role of ^18^F-fluoro-D-deoxyglucose PET and PET/CT in spinal infections. Eur Spine J.

[24] Shemesh S, Kosashvili Y, Groshar D (2015). The value of 18-FDG PET/CT in the diagnosis and management of implant-related infections of the tibia: a case series. Injury Int J Care Injured.

[25] Koort JK, Mäkinen TJ, Suokas E (2005). Efficacy of ciprofloxacin-releasing bioabsorbable osteoconductive bone defect filler for treatment of experimental osteomyelitis due to Staphylococcus aureus. Antimicrob Agents Chemother.

[26] Sugawara Y, Gutowski TD, Fisher SJ (1999). Uptake of positron emission tomography tracers in experimental bacterial infections: a comparative biodistribution study of radiolabeled FDG, thymidine, L-methionine, 67Ga-citrate, and 125I-HSA. Eur J Nucl Med.

[27] Brown TL, Spencer HJ, Beenken KE (2012). Evaluation of dynamic [18F]-FDG-PET imaging for the detection of acute post-surgical bone infection. PLoS One.

[28] Koort JK, Ma¨kinen TJ, Knuuti J (2004). Comparative 18F-FDG PET of experimental *Staphylococcus aureus* osteomyelitis and normal bone healing. J Nucl Med.

[29] Odekerken JC, Walenkamp GH, Brans BT (2014). The longitudinal assessment of osteomyelitis development by molecular imaging in a rabbit model. Biomed Res Int.

[30] Haddad FS, Muirhead-Allwood SK, Manktelow AR (2000). Two stage uncemented revision hip arthroplasty for infection. J Bone Jt Surg.

[31] Walcott BP, Redjal N, Coumans JV (2012). Infection following operations on the central nervous system: deconstructing the myth of the sterile field. Neurosurg Focu.

[32] Calhoun JH, Mader JT (1997). Treatment of osteomyelitis with a biodegradable antibiotic implant. Clin Ortho.

[33] Garvin KL, Miyano JA, Robinson D (1994). Polylactide/polyglycolide antibiotic implants in the treatment of osteomyelitis: a canine model. J Bone Joint Surg Am.

[34] Zhang X, Wyss UP, Pichora D, Goosen MF (1994). Biodegradable controlled antibiotic release devices for osteomyelitis: optimization of release properties. J Pharm Pharmacol.

[35] Jacob E, Setterstrom JA, Bach DE (1991). Evaluation of biodegradable ampicillin anhydrate microcapsules for local treatment of experimental staphylococcal osteomyelitis. Clin Ortho.

[36] De Winter F, Vogelaers D, Gemmel F (2002). Promising role of 18-F-fluoro-D-deoxyglucose positron emission tomography in clinical infectious diseases. Eur J Clin Microbiol Infect Dis.

[37] Zhuang HM, Alavi A (2002). 18-Fluorodeoxyglucose positron emission tomographic imaging in the detection and monitoring of infection and inflammation. Semin Nucl Med.

[38] Paik JY, Lee KH, Choe YS (2004). Augmented 18F-FDG uptake in activated monocytes occurs during the priming process and involves tyrosine kinases and protein kinase C. J Nucl Med.

[39] Mochizuki T, Tsukamoto E, Kuge Y (2001). FDG uptake and glucose transporter subtype expressions in experimental tumor and inflammation models. J Nucl Med.

[40] Pauwels EKJ, Ribeiro MJ, Stoot JH (1998). FDG accumulation and tumor biology. Nucl Med Biol.

[41] Meyer M, Gast T, Raja S (1994). Increased F-18 FDG accumulation in an acute fracture. Clin Nucl Med.

[42] Kubota R, Yamada S, Kubota K (1992). Intratumoral distribution of fluorine-18-fluorodeoxyglucose in vivo: high accumulation in macrophages and granulation tissue studied by microautoradiography. J Nucl Med.

[43] Chatziioannou S, Papamichos O, Gamaletsou MN (2015). 18-Fluoro-2-deoxy-D-glucose positron emission tomography/computed tomography scan for monitoring the therapeutic response in experimental Staphylococcus aureus foreign-body osteomyelitis. J Orthop Surg Res.

[44] Lankinen P, Lehtimäki K, Hakanen AJ (2012). A comparative 18F-FDG PET/CT imaging of experimental Staphylococcus aureus osteomyelitis and Staphylococcus epidermidis foreign-body-associated infection in the rabbit tibia. EJNMMI Res.

[45] Zhuang H, Duarte PS, Pourdehnad M (2001). The promising role of 18F-FDG PET in detecting infected lower limb prosthesis implants. J Nuc Med.

